# Supplementary material for: Strain diversity drives heterogeneous responses to tuberculosis combination therapy
Source: bioRxiv. 2025 Oct 6:2025.10.06.680645. Preprint. [Version 1] doi: 10.1101/2025.10.06.680645 (PMC12632628; doi:10.1101/2025.10.06.680645)
Supplement: Supplement 1 [file NIHPP2025.10.06.680645v1-supplement-1.pdf]

bioRxiv preprint doi: https://doi.org/10.1101/2025.10.06.680645; this version posted October 6, 2025. The copyright holder for this preprint (which was not certified by peer review) is the author/funder, who has granted bioRxiv a license to display the preprint in perpetuity. It is made available under aCC-BY 4.0 International license.

Appendix 1

Supplementary Methods

Antibiotics preparation and dispensing

Each antibiotic was diluted in dimethyl sulfoxide (DMSO) to create a stock solution, which was then prepared into single-use aliquots and stored at -20°C until use. Dispensing errors were avoided with the use of the HP D300e digital dispenser to dispense drugs and drug combinations into 384-well plates, and well positions were randomized to control for plate effects.

In vitro model acclimation

Mtb were acclimated to cholesterol-supplemented medium as follows. A base medium containing 7H9 powder (4.7g/L), NaCl (100mM), tyloxapol (0.05%), and fatty acid-free BSA (0.5g/L) was prepared and heated to 37°C, and a cholesterol stock solution was prepared by dissolving cholesterol in a 1:1 mixture of ethanol and tyloxapol and heated to 80°C for 30 minutes. This mixture was then added to pre-warmed base medium at a final concentration of 0.2 mM. Mtb were grown in standard medium to mid-log phase and were sub-cultured once prior to acclimation. Once acclimated to cholesterol medium, cultures were sub-cultured once into fresh medium and grown to mid-log phase prior to drug treatment assays.

Growth rate inhibition (GR) metrics calculation

GR values were derived using the following equation[30]:

$$\mathrm{GR(c)} = 2^{\frac{log_2(\frac{x(c)}{x_0})}{log_2(\frac{x_{ctrl}}{x_0})}} - 1$$

Where x(c) is the OD_{600} value of a drug-treated sample at concentration c at a specific time point, x_{ctrl} is the measurement of the untreated control at the same time point, and x_0 is the OD_{600} value measured immediately prior to drug exposure (time point 0). GR values quantify the relative growth rate of treated cells compared with untreated cells.

GR was calculated for each drug concentration in the dose–response curve, and a three-parameter Hill function was fit to the data:

$$\mathrm{GR(c)} = \mathrm{GR_{inf}} + \frac{1 - \mathrm{GR_{inf}}}{1 + (\frac{c}{\mathrm{EC}_{50}^{GR}})^{h_{\mathrm{GR}}}}$$

Where $\mathrm{GR_{inf}}$ denotes the maximal inhibitory effect, EC_{50}^{GR} is the concentration at half-maximal growth rate inhibition, h_{GR} is the Hill slope, and c is the concentration of the drug

bioRxiv preprint doi: https://doi.org/10.1101/2025.10.06.680645; this version posted October 6, 2025. The copyright holder for this preprint (which was not certified by peer review) is the author/funder, who has granted bioRxiv a license to display the preprint in perpetuity. It is made available under aCC-BY 4.0 International license.

supplied. $\mathrm{GR_{max}}$ is the maximum effect of a drug at its highest tested concentration and ranges from -1 to 1. A negative value corresponds to a cytotoxic response, a value of 0 corresponds to a cytostatic response, and a positive value corresponds to partial growth inhibition.

By definition, GRmax values range from -1 (complete cytotoxicity) to 1 (no inhibition). In cholesterol, where the doubling times for x_{ctrl} can exceed 90h for some strains, the denominator in the GR equation, $log_2(\frac{x_{ctrl}}{x_0})$ becomes small, making the metric sensitive to minor sources of error such as background noise. Additionally, cholesterol often precipitates over the course of the 12-day incubation step and can increase apparent OD_{600} readings. These effects can occasionally produce computed GR values slightly below -1, which are not biologically possible (as they imply more than 100% loss of cells) and therefore represent numerical artifacts rather than true phenotypes. We therefore bounded all values less than -1 to -1, preserving the intended scale of GR and preventing spurious extreme values from influencing downstream analyses.

Drug interaction quantification

For drug combinations, drug interactions were quantified using Loewe additivity as the null model to compute fractional inhibitory concentration (FIC) values. For each drug combination, the expected IC value was calculated by summing the individual IC values of each constituent drug. The FIC value was then computed as the ratio of the experimentally observed IC value of the drug combination to the expected IC value. FIC values were log_2-transformed to center synergy and antagonism scores at 0. A negative log_2FIC value indicates synergy, a positive log_2FIC value indicates antagonism, and a log_2FIC value at zero indicates additivity. FIC values were calculated at IC_{50} (FIC_{50}).

bioRxiv preprint doi: https://doi.org/10.1101/2025.10.06.680645; this version posted October 6, 2025. The copyright holder for this preprint (which was not certified by peer review) is the author/funder, who has granted bioRxiv a license to display the preprint in perpetuity. It is made available under aCC-BY 4.0 International license.

Supplementary Tables and Figures

Drug	Abbreviation	Mechanism of action
Bedaquiline	BDQ	Respiration
Telacebec	Q203	Respiration
Pyrazinamide	PZA	Respiration or Multi–effect
Isoniazid	INH	Cell wall
SQ109	SQ109	Cell wall
TBA–7371	TBA	Cell wall
Moxifloxacin	MOX	DNA
Rifampicin	RIF	RNA
Linezolid	LIN	Protein
Pretomanid	PRE	Multi–effect

Supplementary Table 1 Names, abbreviations, and mechanisms of action of the ten antibiotics used in this study.

For all ten antibiotics, the commercial name, the abbreviation used in this study, and its mechanism of action are listed in the left-most, middle, and right-most columns, respectively.

bioRxiv preprint doi: https://doi.org/10.1101/2025.10.06.680645; this version posted October 6, 2025. The copyright holder for this preprint (which was not certified by peer review) is the author/funder, who has granted bioRxiv a license to display the preprint in perpetuity. It is made available under aCC-BY 4.0 International license.

Single drugs	Corresponding drug pairs
BDQ	BDQ + PRE
	BDQ + SQ109
	BDQ + LIN
	BDQ + INH
	BDQ + MOX
	BDQ + RIF
	BDQ + Q203
	BDQ + TBA
	BDQ + PZA
PRE	PRE + BDQ
	PRE + SQ109
	PRE + LIN
	PRE + INH
	PRE + MOX
	PRE + RIF
	PRE + Q203
	PRE + TBA
	PRE + PZA
SQ109	SQ109 + BDQ
	SQ109 + PRE
	SQ109 + LIN
	SQ109 + INH
	SQ109 + MOX
	SQ109 + RIF
	SQ109 + Q203
	SQ109 + TBA
	SQ109 + PZA
LIN	LIN + BDQ
	LIN + PRE
	LIN + SQ109

bioRxiv preprint doi: https://doi.org/10.1101/2025.10.06.680645; this version posted October 6, 2025. The copyright holder for this preprint (which was not certified by peer review) is the author/funder, who has granted bioRxiv a license to display the preprint in perpetuity. It is made available under aCC-BY 4.0 International license.

Single drugs	Corresponding drug pairs
	LIN + INH
	LIN + MOX
	LIN + RIF
	LIN + Q203
	LIN + TBA
	LIN + PZA
INH	INH + BDQ
	INH + PRE
	INH + SQ109
	INH + LIN
	INH + MOX
	INH + RIF
	INH + Q203
	INH + TBA
	INH + PZA
MOX	MOX + BDQ
	MOX + PRE
	MOX + SQ109
	MOX + LIN
	MOX + INH
	MOX + RIF
	MOX + Q203
	MOX + TBA
	MOX + PZA
RIF	RIF + BDQ
	RIF + PRE
	RIF + SQ109
	RIF + LIN
	RIF + INH
	RIF + MOX
	RIF + Q203

bioRxiv preprint doi: https://doi.org/10.1101/2025.10.06.680645; this version posted October 6, 2025. The copyright holder for this preprint (which was not certified by peer review) is the author/funder, who has granted bioRxiv a license to display the preprint in perpetuity. It is made available under aCC-BY 4.0 International license.

Single drugs	Corresponding drug pairs
	RIF + TBA
	RIF + PZA
Q203	Q203 + BDQ
	Q203 + PRE
	Q203 + SQ109
	Q203 + LIN
	Q203 + INH
	Q203 + MOX
	Q203 + RIF
	Q203 + TBA
	Q203 + PZA
TBA	TBA + BDQ
	TBA + PRE
	TBA + SQ109
	TBA + LIN
	TBA + INH
	TBA + MOX
	TBA + RIF
	TBA + Q203
	TBA + PZA
PZA	PZA + BDQ
	PZA + PRE
	PZA + SQ109
	PZA + LIN
	PZA + INH
	PZA + MOX
	PZA + RIF
	PZA + Q203
	PZA + TBA

Supplementary Table 2 Corresponding drug pairs of each individual drug used in this study.

All ten antibiotics and all nine corresponding drug pairs of each individual antibiotic are listed on the left-most and right-most columns, respectively.

bioRxiv preprint doi: https://doi.org/10.1101/2025.10.06.680645; this version posted October 6, 2025. The copyright holder for this preprint (which was not certified by peer review) is the author/funder, who has granted bioRxiv a license to display the preprint in perpetuity. It is made available under aCC-BY 4.0 International license.

Three-way combinations
BDQ + PRE + LIN
BDQ + PRE + INH
BDQ + PRE + MOX
BDQ + PRE + RIF
BDQ + LIN + INH
BDQ + LIN + MOX
BDQ + LIN + RIF
BDQ + INH + MOX
BDQ + INH + RIF
BDQ + MOX + RIF
PRE + LIN + INH
PRE + LIN + MOX
PRE + LIN + RIF
PRE + INH + MOX
PRE + INH + RIF
PRE + MOX + RIF
LIN + INH + MOX
LIN + INH + RIF
LIN + MOX + RIF
INH + MOX + RIF

Supplementary Table 3 All three-way combinations tested in this study.

bioRxiv preprint doi: https://doi.org/10.1101/2025.10.06.680645; this version posted October 6, 2025. The copyright holder for this preprint (which was not certified by peer review) is the author/funder, who has granted bioRxiv a license to display the preprint in perpetuity. It is made available under aCC-BY 4.0 International license.

Strain	SRA accession number
044	SRR5073757
070	SRR5074078
082	SRR5073981
084	SRR5073991
139	SRR5073977
185	SRR5073510
245	SRR5067510
355	SRR5067598
358	SRR5067503
414	SRR5067585
478	SRR5065461
545	SRR5065319
617	SRR5065320
Erdman	SRR24083712

Supplementary Table 4 Identification and accession numbers of the 14 strains used in this study.

For all 14 strains used in this study, the identification name and Sequence Read Archive (SRA) accession numbers are listed in the left and right columns, respectively.

bioRxiv preprint doi: https://doi.org/10.1101/2025.10.06.680645; this version posted October 6, 2025. The copyright holder for this preprint (which was not certified by peer review) is the author/funder, who has granted bioRxiv a license to display the preprint in perpetuity. It is made available under aCC-BY 4.0 International license.

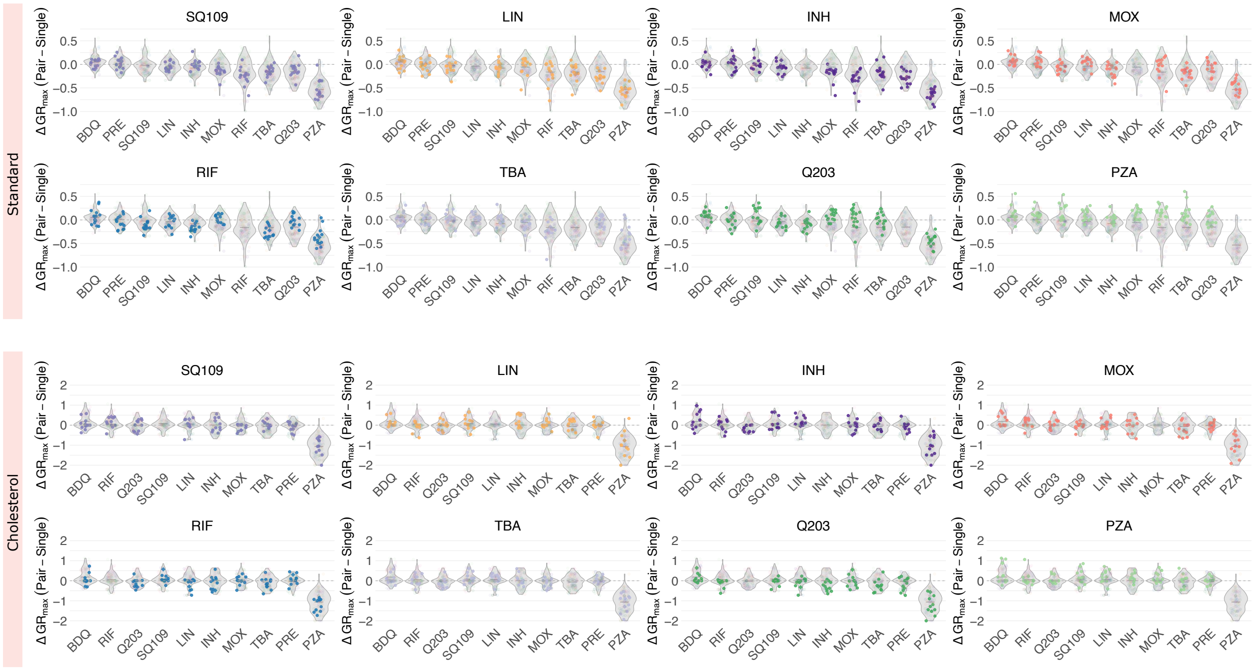

Supplementary Figure 1 Comparison of GR_{max} values between single drugs and drug pairs.

In both standard (top) and cholesterol (bottom) conditions, each of the eight panels represents the change in GR_{max} between a single drug and its 9 corresponding pairs. ΔGR_{max} is calculated by subtracting the GR_{max} value of the single drug from that of a corresponding drug pair. For each single drug, all nine corresponding drug pairs were tested in fourteen clinical isolates in standard and eleven clinical isolates in cholesterol. Each point denotes a GR_{max} value of a drug pair for an individual isolate and represents the median GR_{max} value across three biological replicates. A negative ΔGR_{max} value indicates an improvement in GR_{max} when a single drug is combined with another drug.

bioRxiv preprint doi: https://doi.org/10.1101/2025.10.06.680645; this version posted October 6, 2025. The copyright holder for this preprint (which was not certified by peer review) is the author/funder, who has granted bioRxiv a license to display the preprint in perpetuity. It is made available under aCC-BY 4.0 International license.

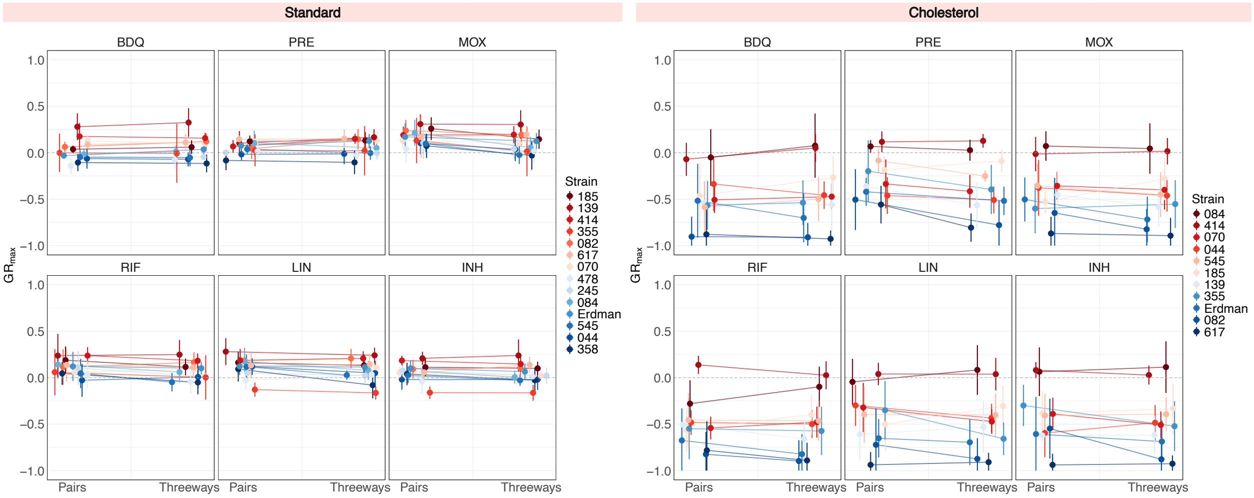

Supplementary Figure 2 Comparison of GR_{max} values between drug pairs and three-way combinations.

In both standard (left) and cholesterol (right) conditions, each of the six panels represents a single drug, with the mean GR_{max} value of its 5 corresponding drug pairs on the left side and the mean GR_{max} value of its ten corresponding three-way combinations on the right side. Vertical bars represent the standard deviation of the mean GR_{max} value. Each point corresponds to one of 14 clinical isolates in standard (left), or one of 11 clinical isolates in cholesterol (right).

bioRxiv preprint doi: https://doi.org/10.1101/2025.10.06.680645; this version posted October 6, 2025. The copyright holder for this preprint (which was not certified by peer review) is the author/funder, who has granted bioRxiv a license to display the preprint in perpetuity. It is made available under aCC-BY 4.0 International license.

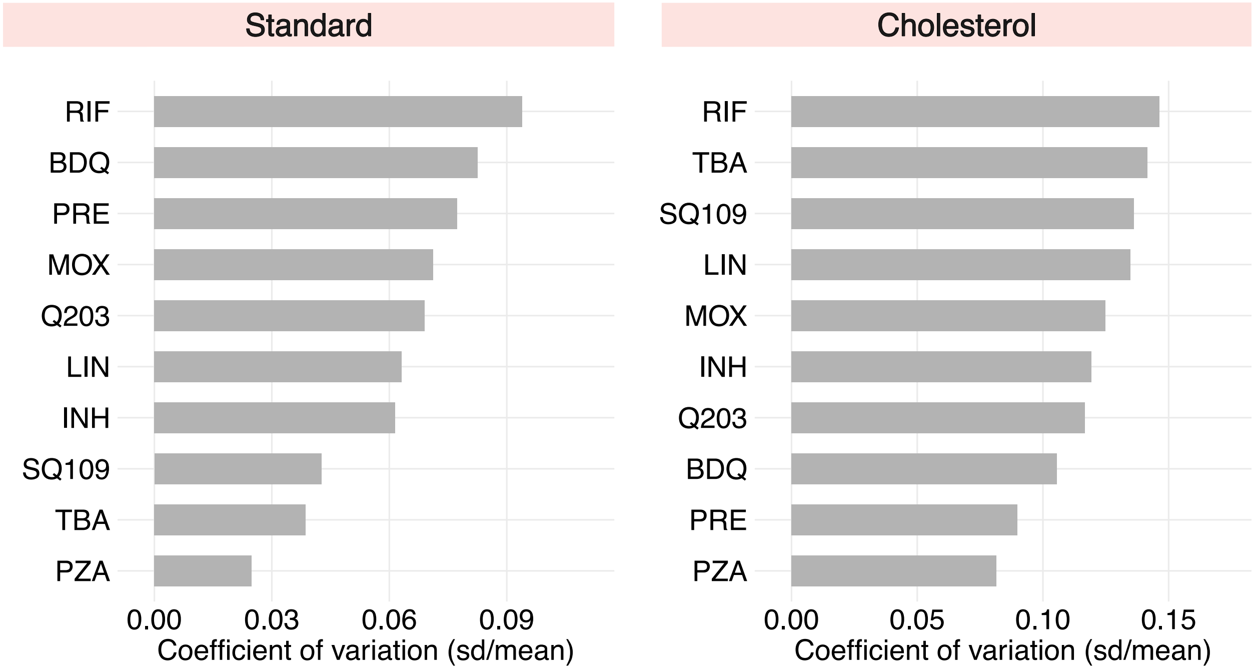

Supplementary Figure 3 Coefficient of variation (CV) values of ten single drugs

In both standard (left) and cholesterol (right) conditions, the CV was calculated for each drug by dividing the standard deviation of the GR_{max} values by the mean GR_{max} values of 14 isolates in standard and 11 isolates in cholesterol. Drugs were rank ordered from largest to smallest CV (top to bottom, respectively), with larger values indicating greater variation in GR_{max} between strains.

bioRxiv preprint doi: https://doi.org/10.1101/2025.10.06.680645; this version posted October 6, 2025. The copyright holder for this preprint (which was not certified by peer review) is the author/funder, who has granted bioRxiv a license to display the preprint in perpetuity. It is made available under aCC-BY 4.0 International license.

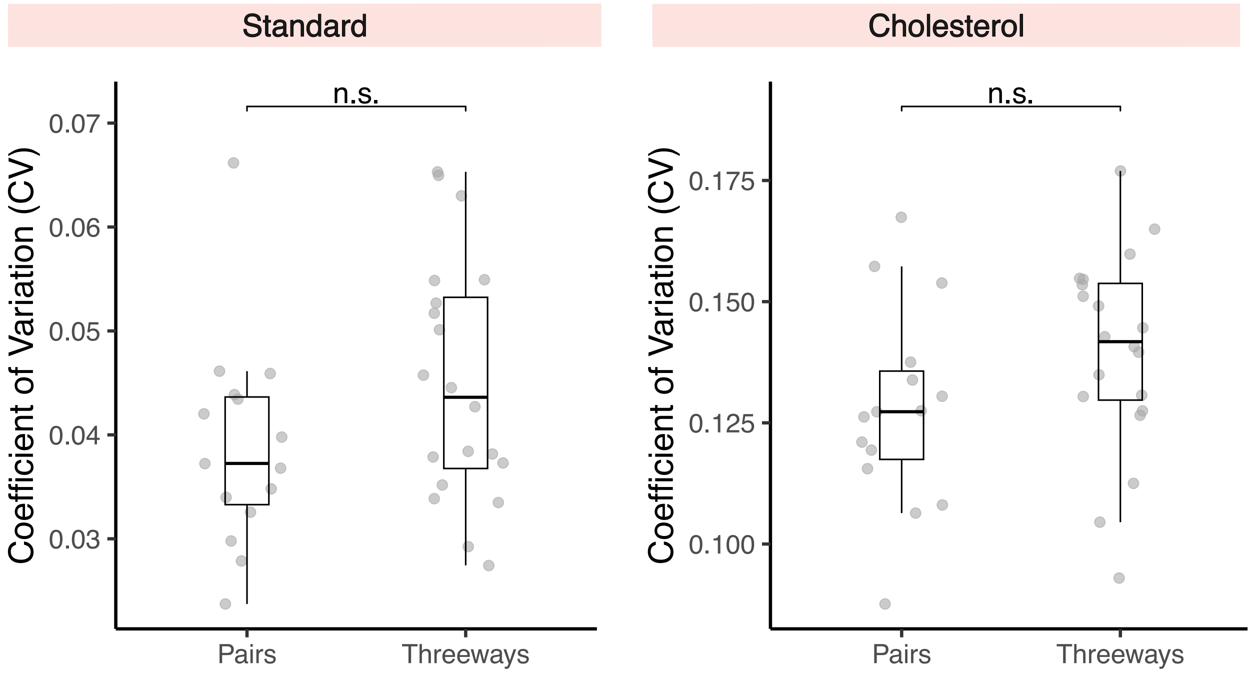

Supplementary Figure 4 Comparison of mean coefficient of variation (CV) between drug pairs and three-way combinations.

In both standard (left) and cholesterol (right) conditions, the CV of each drug pair (total of 15) and three-way combinations (total of 20) was calculated by dividing the standard deviation of the GR_{max} values by the mean GR_{max} values of 14 isolates in standard and 11 isolates in cholesterol. A Student's t-test was performed to determine whether the difference between the mean CV of drug pairs and the mean CV of three-way combinations is statistically significant.
